# The Stars of Night

**Published:** 1870-04

**Authors:** 


### THE STARS OF NIGHT.

There are certain associations between love and "moonshiny" nights.

> "No baleful star
> Darts his malignant fires so far,"

is one of the rhymes of "Isaac Watts," whose name will never die.

"Lunatics" have been supposed for ages to owe their unhappy condition to certain malign influences of "Luna," the Roman name for moon.

"Star-gazers" have long been considered among the crazy folk. But we can meditate about the stars without becoming demented.

If a wagon wheel is started at the head of Broadway, and turns and turns until it reaches the Battery, its rim and hub turn round the axle, but at the end the axle and the wheel are several miles distant from the place of starting; meanwhile the houses seen at Union Square, where the wheel started, have disappeared, while others meet the eye at Whitehall, where Broadway ends, which were not seen before.

The sun is the hub of this world, and both sun and world have been revolving for thousands of years in space, but both, like the wheel, are far distant from their starting-point. Where are they going? What is the end of their journey? Some stars which used to be seen are seen no more: they answer to the houses seen at the head of Broadway.

Other stars are appearing which were never before seen by mortal eyes: they answer to the houses at the foot of Broadway.

But philosophers have been fond of explanations like the following:—

The stars which have disappeared have been burned up, and are the wastes of immensity.

German astronomers assert that two stars — one in the constellation of "Corona," and the other the "Ætha Argus" (generally marked on astronomical charts by the Greek letter H) have taken fire.

The new stars which appear are those which were created at such an immense distance that the light emanating from them at the rate of many millions of miles a year has just reached this world. The very idea almost overwhelms us, as faintly shadowing the immensity of space and the inconceivable greatness of the Architect of all these worlds.

Two hundred and fifty thousand stars have been observed and distinctly mapped out. Ten of the largest observatories in the world, of which that at Chicago is one, are now engaged in fixing the exact position of these stars; it will require ten years to complete the sublime work, or twenty-five thousand stars for each observatory, the object being to decide in what direction each star has proceeded through illimitable space.

We know that angels are employed by the Almighty mind to perform certain duties throughout the kingdoms of this world as "aids" are employed by generals on the battle-field, to carry out instructions, and, as it were, to bring back information; in performing these duties they are sometimes behind time, they do not get through their work as soon as calculated upon, then they have to help one another, or give a satisfactory excuse. We are now speaking after the manner of men. In the book of Daniel, in the Old Testament, these statements are verified in reference to the Prince of the Power of Persia. What has been said is certainty, but now we enter the fields of unsatisfactory conjecture. May not the employment of the redeemed in heaven be in part as messengers of the Holy One, journeying from star to star, from sun to sun, from system to system, drinking in draughts of knowledge, instruction, information, rising higher and higher towards the likeness of the Infinite.

This human life of ours is

THE SCHOOL-HOUSE OF THE SKIES.

We should improve our time, our talents, and our opportunities to the very best advantage. But what years of time are lost by unnecessary sickness, unnecessary because avoidable; and are we not living to purpose in teaching people how to live so as to avoid disease, and thus give them more time for the schooling referred to, and not only so, but by having good health to enable them to study to greater advantage, with a clearer brain, and with a more vigorous intellect.

### ASYMPTOTE OF THE HYPERBOLA.

We were once travelling in a foreign land, a land of savages, a wilderness country abounding with savage beasts of prey, fierce, bloody, and relentless. Night was coming on, and the narrow pathway became more and more indistinct. Our only companion was a young physician, both of us fresh from the medical university. Delighting in human bones, we were in search of a battle-field, where slaughtered multitudes had slept the sleep of the warrior. Coming to a point where the path seemed to diverge as if it would meet again a few yards ahead, I took the right, he the left. We never met again; every step took us farther apart. To-day, eternity divides us. This is not the asymptote of the hyperbola.

Suppose we had started at the other end of this diverging. He starting upon the one fork and I the other, every step would have brought us nearer and nearer until we should have met. Readers, this is not the asymptote of the hyperbola; it is just as far from it as the other. But now that you and I are both in a good humor, and are at leisure, let me ask you a plain, simple question. Would you like to know what the asymptote of the hyperbola is? I always had a great delight in making abstruse things as plain as a pike-staff — when it could be done. One time, an age ago, we were fairly stumped when the minister said "Anthropomorphism is theopneustic." We have studied that tremendous assertion, off and on, for nearly thirty years, and have not yet got the hang of it; but we intend to keep at it; light will break in some of these times.

But now having waked you up, to keep you from gaping, let us go back to that hyperbola, and see what can be made out of it.

When my companion and I separated at that little fork of the road, every step took us farther and farther apart; if we had begun at the other end of the fork, every step would have brought us nearer together; now the asymptote of the hyperbola is neither the one nor the other; nor is it between one or the other, but it is between them both, all mixed up, for it is as two men travelling, each step bringing them nearer and nearer each other, but so slowly that travelling eternally they would never meet; that is the asymptote of the hyperbola getting near and nearer in two straight lines, but never meeting. So that boundless as the space is of which we were a while ago speaking, it would not be extensive enough to allow a meeting of the two lines forever approaching, but meeting never.

Duration is as eternal as space is illimitable. We inhabit that space, we have entered upon that duration, but in a sense we enter upon that duration more perfectly, with higher capabilities, because unclogged by materialities. The soul, the man himself, the Ego, the I myself, will be free beyond the grave; free to study, free to acquire knowledge, free to rise in all that is pure, and glorious, and good; rising forever; forever nearing the character, the intelligence, the goodness, of the Infinite One; never attaining, but always adding, acquiring, approaching, — these acquisitions, whether of knowledge or goodness being happiness itself. This is the bliss of immortality, loving more and more that which we are approaching, the likeness of, that is, the wisdom and holiness of the great Father, Maker, and Saviour of us all, this is the moral asymptote of the hyperbola.

If we all could spend more of our daily time in contemplating these truths, we should live longer, because such thoughts have a soothing effect on the mind; they are like oil upon the troubled waters of life; they still the tempests of the mind, they put out the fires which burn us up prematurely, and give us that equability of mental and physical emotion and motion which enables the mortal machine to work equably, smoothly, leisurely, and long; as proof, natural philosophers, men who spend most of their lives in contemplating the great natural and spiritual truths which are presented to the mind in its investigations of what God has made, live the longest.

